# Alpinetin activates the δ receptor instead of the κ and μ receptor pathways to protect against rat myocardial cell apoptosis

**DOI:** 10.3892/etm.2013.1359

**Published:** 2013-10-23

**Authors:** CHUANTAO SUO, LIBO SUN, SHUANG YANG

**Affiliations:** 1 Department of Cardiology, Daqing General Hospital Group Oilfield General Hospital, Daqing, Heilongjiang 163000, P.R. China; 2 Department of Gastrointestinal Surgery, China-Japan Union Hospital, Jilin University, Changchun, Jilin 130021, P.R. China; 3 Department of Cardiology, Second Affiliated Hospital of Harbin Medical University, Harbin, Heilongjiang 150001, P.R. China

**Keywords:** alpinetin, δ receptor, cardiomyocytes, apoptosis, PKC/ERK pathways

## Abstract

Alpinetin is a natural flavonoid that protects cells against fatal injury in ischemia-reperfusion. δ receptor activation protects myocardial cells from trauma; however, the mechanism is unknown. The aim of this study was to explore the function of alpinetin in δ receptor-mediated myocardial apoptosis. The myocardial cells of newly born rats were cultivated and myocardial apoptosis was induced by serum deprivation. The MTT method was used to evaluate cell viability and Annexin V-fluorescein isothiocyanate (FITC)/propidium iodide (PI) staining was used to analyze apoptosis. The expression levels of opioid receptor mRNA and protein were tested using reverse transcription-polymerase reaction (RT-PCR) and western blot assays. In addition, an opioid receptor antagonist, as well as protein kinase C (PKC) and extracellular signal-regulated kinase (ERK) inhibitors, were used to determine the inferred signaling pathway. The results showed that that alpinetin reduced the myocardial apoptosis induced by serum deprivation in a concentration-dependent manner. However, the protection conferred to the myocardial cells by alpinetin was blocked by the δ opioid receptor antagonist naltrindole, as well as by PKC and ERK inhibitors (GF109203X and U0126, respectively). In addition, it was shown that alpinetin was able to maintain the stability of the mitochondrial membrane potential, lower the level of intracytoplasmic cytochrome *c* and reduce Bax displacement from the cytoplasm to the mitochondria. It was concluded that alpinetin was able to activate δ receptors to induce the endogenous protection of myocardial cells via the PKC/ERK signaling pathway.

## Introduction

Since its discovery, apoptosis has been shown to be important in the pathogenesis of many diseases (1,2). Myocardial apoptosis is the main means of myocardial cell death during myocardial ischemia, anoxia and ischemia-reperfusion (3), and is an important cytological factor leading to a number of heart diseases. The reduction of non-physiological myocardial apoptosis is of great significance in the protection of cardiac structure and function.

Alpinetin is a natural flavonoid predominantly found in the ginger family, such as turmeric, cardamom and radix curcumae. In recent years, with the increased study of flavonoids, it has been demonstrated that flavonoids exhibit a number of functions, including exerting antibacterial, antioxidative, anticancer, antithrombotic, antihypertensive, antidiabetic, antiemetic and analgesic effects (4–6), in addition to restraining the growth of tumor cells (7). However, to date, there remains little insight into the function of alpinetin in the growth of normal human myocardial cells. Furthermore, the detailed mechanism of action of alpinetin remains unclear.

Opioid receptors are G-protein-coupled receptors and are extensively distributed throughout the human body. A number of studies have shown that δ receptor activation may promote the proliferation of rat myocardial cells (8,9) and imitate ischemia preconditioning to protect the heart and brain (10,11). As studies of δ receptor functions have continued, it has been shown that the δ receptor is critical in the regulation of the onset and development of cardiac diseases. Aitchison *et al*(12) observed in an *in vitro* rat heart model that preconditioning with (D-Ala^2^, D-Leu^5^)enkephalin (DADLE), a δ receptor agonist, was able to significantly reduce the area of myocardial infarction. Furthermore, the δ receptor blocker naltrindole has been shown to reverse the cardioprotective effects of ischemic preconditioning (IPC) by abolishing the reduction in the myocardial infarction area (13). The results of these studies have indicated that the δ receptor is important in cell growth and proliferation.

Protein kinase C (PKC) is a member of the serine/threonine kinase family, which is widely distributed in the body. Under quiescent conditions, intracellular PKC exists in the cytoplasm in a passivation form. However, when the cell is stimulated, PKC is induced to translocate from the cytoplasm to the cytomembrane to be activated. PKC exhibits extensive biological activity. It has been demonstrated that PKC participates in protection against myocardial ischemia, and that cell proliferation and apoptosis and the regulation of myocardial contraction may induce myocardial hypertrophy, myocardial fibrosis and cardiac failure. The PKC signaling pathway is an important pathway for the proliferation, differentiation and survival of numerous types of cells (14). In recent years, the opioid receptor has been revealed to exhibit the common sequence for PKC phosphorylation. It was observed in an isolated heart model that the protective effect of morphine in reducing the myocardial infarction area was blocked by chelerythrine, a PKC blocker (15). This indicated that PKC induced the protective effects of morphine preconditioning.

As shown in previous studies, the δ receptor, similar to other G-protein-coupled receptors, is able to activate extracellular signal-regulated kinase (ERK), a member of the mitogen-activated protein kinase (MAPK) family (16–20). ERK may participate in the proliferation process of hepatoma cells (21); therefore, it has been inferred that the δ receptor may cause the proliferation of myocardial cells via the ERK signal transduction pathway. A previous study has demonstrated that the activated δ receptor protected PC12 cells via the MEK (MAP kinase kinase)-ERK pathway (22). Furthermore, it has been shown that the activation of the δ receptor in SH-SY5Y cells resulted in the activation of Ca^2+^/calmodulin-dependent protein kinase II (CaMKII) and PKC, which, in turn, activated ERK (23). This indicated that the ERK pathway was also crucial to the proliferation of myocardial cells.

In the current study, the myocardial cells of newly born rats were cultured *in vitro* and myocardial apoptosis was induced by serum deprivation. This was used as a model to investigate the impacts of alpinetin and the δ receptor on myocardial apoptosis and its molecular mechanism.

## Materials and methods

### Animals and experimental reagents

Healthy male Sprague-Dawley (SD) rats, born within the previous seven days, were provided by the Laboratory Animal Center of Jilin University (Changchun, China). All surgical procedures were performed in accordance with the Guide for the Care and Use of Laboratory Animals (1993) and followed the ethical standards. Alpinetin, naltrindole, GF109203X, U0126 and Dulbecco’s modified Eagle’s medium (DMEM) were obtained from Sigma (St. Louis, MO, USA) and fetal calf serum was purchased from Gibco-BRL (Grand Island, NY, USA). The Annexin V-fluorescein isothiocyanate (FITC) kit used in the study was obtained from Bio-Rad (Hercules, CA, USA), while δ, κ and μ opioid receptor antibodies and anti-actin, PKC, ERK, Bcl-2 and Bcl-2-associated X protein (Bax) antibodies were purchased from Santa Cruz Biotechnology, Inc. (Santa Cruz, CA, USA). Cytochrome *c* (Cyt *c)*, caspase-3 and caspase-9 antibodies were purchased from Cell Signaling Technology, Inc. (Danvers, MA, USA). The present study was approved by the Ethics Committee of The Basic Medical College of Jilin University.

### Separation and culture of rat myocardial cells

The hearts of the healthy male SD rats (born within the previous seven days) were extracted via a thoracotomy, washed three times with phosphate-buffered saline (PBS) solution and cut into tissue fragments measuring 1 mm^3^. Following this, trypsin, at a concentration of 0.08%, was added to digest the cells. The digested cells were subsequently suspended in DMEM culture medium containing 15% fetal calf serum and 1% double-antibody, prior to being suspended in culture medium with uniform distribution. Following this, the cells were placed into a CO_2_ incubator containing 5% CO_2_ and 95% oxygen for culture. Rat myocardial cells were cultured for 48 h and the medium of each group, with the exception of the control group, was replaced with serum-free DMEM and cultured further. The cells were collected at different time points (0, 24, 48, and 72 h) for analysis. During serum deprivation, the intervention groups were treated with different concentrations of Alpinetin (0, 40, 80 and 120 mg/ml) for 48 h.

### Assessment of myocardial cell viability

The myocardial cells of the various groups were cultured *in vitro* and treatment factors were administered. The cell density was then adjusted to 1×10^5^ cells/ml, prior to inoculation into 96-well culture plates, with eight complex wells for each group. A total of 20 μl of 5 mg/ml MTT solution was added to each well 4 h prior to testing and then the cells were cultured for a further 4 h in the CO_2_ incubator. Following this, the culture liquid was removed and 0.15 ml dimethylsulfoxide (DMSO) was added to each well, prior to 15 min oscillation being performed. The optical density (OD) value of each well was subsequently measured at a wavelength of 570 nm and the measured light absorption value was converted into the number of cells, in order to determine the cell viability. Cell viability was calculated using the following formula: Cell viability = light absorption value of the experimental group/light absorption value of the control group × 100.

### Analysis of apoptosis using the Annexin V-FITC/propidium iodide (PI) double-labeling method

Trypsin (0.25%) digestion was used to collect the cells of all the experimental groups and the cell density was adjusted to 1×10^6^ cells/ml. A total of 10 μl Annexin V-FITC and 5 ml PI were added, respectively, for 30 min staining at 4°C, prior to analysis being performed using flow cytometry (BD Biosciences, Franklin Lakes, NJ, USA).

### Analysis of mitochondrial membrane potential

The myocardial cells of all the experimental groups were collected and the mitochondria were extracted using the method of Tang *et al*(24). The mitochondrial density in the cell count under a microscope plate was adjusted for the test. Following this, 10 μg/ml JC-1 solution (dissolved in DMSO) was added, fully mixed and incubated in a 5% CO_2_ dark incubator for 30 min at 37°C. The cells were subsequently analyzed using a flow cytometer (BD Biosciences, Franklin Lakes, NJ, USA) with an emission wavelength of 488 nm. Each sample consisted of 1×10^4^ cells. JC-1 monomers and aggregates were respectively visualized using FL1 and FL2 detectors. FL1-H and FL2-H represented the fluorescence intensity of red and green. The quantitative analysis was conducted using CellQuest™ analysis software (BD Biosciences).

### Cell cycle analysis

The myocardial cells in each of the groups were digested with 0.25% trypsin and fixed overnight with 95% cold ethanol at 4°C. Following this, the cells were washed twice with PBS and the cell density was adjusted to 1×10^6^ cells/ml. The final volume was 100 μl. A total of 500 μl DNAStain synthetic dye liquor (with final concentrations of 50 mg/l RNase, 100 mg/l PI and 1 ml/l Triton X-100) was subsequently added and stored for 30 min at room temperature in a dark place. Analysis was conducted using flow cytometry.

### Extraction of total RNA and the reverse-transcription polymerase chain reaction (RT-PCR)

Total RNA of the myocardial cells was extracted in accordance with the instructions of the RNAiso™ Plus kit (Takara Bio, Inc., Shiga, Japan). Subsequent to calculating the RNA concentration, an RT-PCR kit (Takara Bio, Inc.) was used to perform the RT-PCR, in accordance with the manufacturer’s instructions. Primers for the δ, μ and κ opioid receptors (DOR, MOR and KOR, respectively) and β-actin were synthesized by Invitrogen Life Technologies (Carlsbad, CA, USA) and were as follows: DOR, forward primer 5′-GTTCGGAGAGCTGCTGTGC-3′ and reverse primer 5′-ATTGATGTCCACCAGCGTCC-3′; MOR, forward primer 5′-GCCCTCTACTCTATCGTGTGT-3′ and reverse primer 5′-GGAAACAGTCTGGAAAGTGGT-3′; KOR, forward primer 5′-CGTCTGCTACACCCTGATGATC-3′ and reverse primer 5′-CTCTCGGGAGCCAGAAAGG-3′; β-actin, forward primer 5′-CTGGGACGACATGGAGAAAA-3′ and reverse primer 5′-AAGGAAGGCTGGAAGAGTGC-3′. The reaction conditions for the 50-μl PCR reaction system were as follows: 94°C for 2 min, 94°C degeneration for 30 sec, 60°C annealing for 30 sec and 72°C extension for 30 sec (total, 30 cycles). The PCR products were analyzed using 1.0% agarose gel electrophoresis (AGE) and were scanned and analyzed with a gel imaging system (G:BOX Chemi XR5; Syngene, Cambridge, UK).

### Western blot analysis

The myocardial cells of all the groups were collected and washed twice with PBS, prior to 2 ml lysis solution (Sigma) being added for cell lysis. Once the protein concentration had been determined using the bicinchoninic acid (BCA) method, the protein was stained with bromophenol blue. Identical amounts of protein were added to each well, prior to the proteins being separated with 10% sodium dodecyl sulfate-polyacrylamide gel electrophoresis (SDS-PAGE). The proteins were subsequently transferred to a polyvinylidene difluoride (PVDF) membrane using the semi-dry method and sealed with 5% skimmed mild powder overnight. On the following day, the membrane was washed with Tris-buffered saline and Tween 20 (TBST) and the primary antibody [PKC antibody (1:300), ERK antibody (1:500), Bcl-2 and Bax antibody (1:700), Cyt C antibody (1:200), δ, μ, κ opioid receptor antibody (1:400)] was added. This was incubated for 2 h, prior to bleaching with TBST. The secondary antibody (goat anti-rabbit and goat anti-mouse) was then added and incubated for 2 h. A chemiluminescent reagent was added prior to the capturing of images by exposure of an X-ray film and strip scanning. A gray scale analysis was conducted and β-actin was used for standardization.

### Statistical analysis

SPSS 16.0 statistical software (SPSS, Inc., Chicago, IL, USA) was used for the statistical analysis. The values are shown as the mean ± standard deviation. The statistical analysis was performed using the Student’s t-test, and P<0.05 was considered to indicate a statistically significant difference.

## Results

### Rat myocardial cell apoptosis induced by serum deprivation is attenuated by alpinetin in a concentration-dependent manner

**Figure 1:**
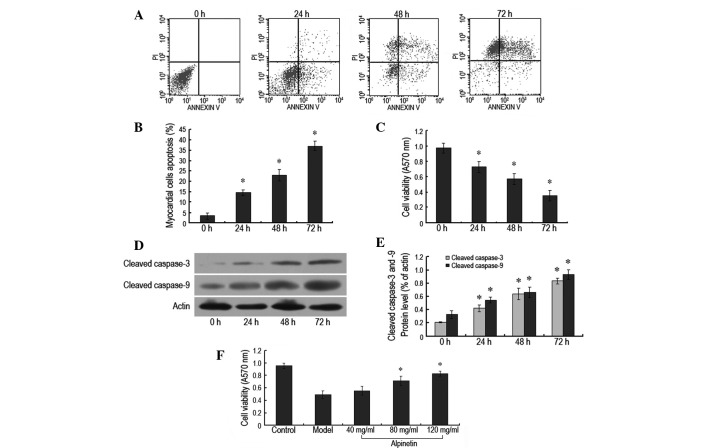
(A and B) Rat myocardial apoptosis, analyzed using flow cytometry; (C) rat myocardial cell viability, assessed using the MTT method and (D and E) western blot analysis of cleaved caspase-3 and cleaved caspase-9 protein expression levels following 0, 24, 48 and 72 h of serum deprivation in rat myocardial cells. (F) MTT analysis of rat myocardial cell viability following the administration of alpinetin at various concentrations (40, 80 and 120 mg/ml) at the same time as serum deprivation, following incubation for 48 h. Data shown are representative results from at least three independent experiments. ^*^P<0.05, versus control cells.

In this experiment, the myocardial cell culture medium was serum-deprived and flow cytometry and the MTT method were used to analyze apoptosis and cell viability, respectively. The cell apoptosis rate of the myocardial cells was demonstrated to be 14.54, 23.18 and 37.11% following 24, 48 and 72 h of serum deprivation, respectively, which was significantly higher than that of the control group (P<0.05; Fig. 1A and B). The A570 nm values of the serum-deprived cells were 0.73±0.17, 0.57±0.17 and 0.35±0.16, respectively, which were also significantly lower than those of the control group (P<0.05; Fig. 1C). In addition, it was observed that the levels of cleaved caspase-3 and cleaved caspase-9 proteins increased as the duration of serum deprivation progressed, which was consistent with the results mentioned previously (Fig. 1D and E). This indicated that serum deprivation was able to simulate *in vivo* ischemia and anoxia to result in rat myocardial apoptosis. Alpinetin, at various concentrations, was administered to the rat myocardial cells at the same time as the serum deprivation. The A570 nm values of the myocardial cells treated with alpinetin were observed to be increased compared with those for the cells undergoing serum deprivation only. As the concentration of alpinetin increased from 40 to 120 mg/ml, the A570 nm values of the rat myocardial cells increased in a concentration-dependent manner. This indicated that alpinetin conferred protection against the rat myocardial apoptosis induced by serum deprivation in a concentration-dependent manner, with the most notable effects apparent when the concentration was 120 mg/ml (Fig. 1F).

### Alpinetin protects myocardial cells via activation of the δ receptor

**Figure 2:**
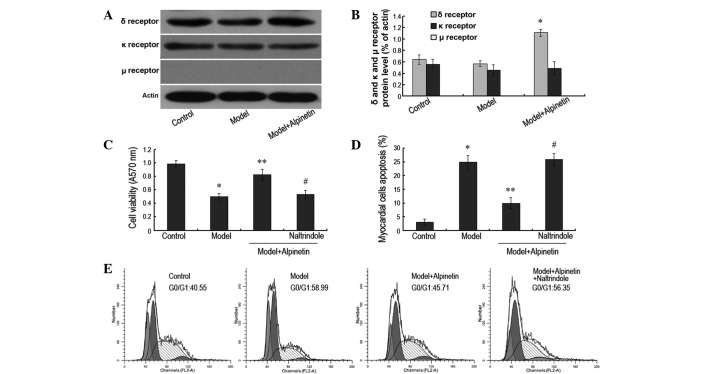
(A and B) Expression levels of δ, κ and μ receptors in rat myocardial cells, as analyzed with western blotting 48 h subsequent to the serum deprivation of rat myocardial cells with simultaneous administration of 120 mg/ml alpinetin. ^*^P<0.05, versus control cells. (C) MTT assay results of rat myocardial cell viability and (D and E) flow cytometry analysis of rat myocardial apoptosis and cell cycle following administration of the δ receptor antagonist naltrindole (10 μM) at the same time as alpinetin administration and serum deprivation. Data shown are the representative results from at least three independent experiments. ^*^P<0.05, versus control group; ^**^P<0.05, versus model group; ^#^P<0.05, versus model + alpinetin group.

As indicated by previous studies, the activation of the δ receptor may promote the proliferation of myocardial cells and protect them to a certain extent (8,9). In this experiment, following the administration of a therapeutic concentration of alpinetin, western blotting revealed the expression level of the δ receptor to have increased significantly in the rat myocardial cells. However, there were no apparent changes in the expression levels of the κ and μ receptors (Fig. 2A and B). When the δ receptor antagonist naltrindole was also administered, it was observed that the myocardial apoptosis rate increased significantly (P<0.05) and the A570 value of the myocardial cell decreased markedly (P<0.05), in comparison with those of the cells treated with alpinetin only (Fig. 2C and D). In addition, cell cycle analysis by flow cytometry demonstrated that the percentage of the myocardial cells in the G0/G1 phase increased significantly following serum deprivation (P<0.05), while the percentage decreased significantly following the administration of 120 mg/ml alpinetin (P<0.05). However, when the δ receptor antagonist was also administered, the percentage of the myocardial cells in G0/G1 phase increased again (P<0.05; Fig. 2E). These results indicate that the protective effect of alpinetin on the myocardial cells was dependent on the activation of the δ receptor, and that this was closely associated with the inhibition of apoptosis and the promotion of cell proliferation and the cell cycle.

### Protection of rat myocardial cells by alpinetin is mediated by the PKC/ERK signaling pathway

**Figure 3:**
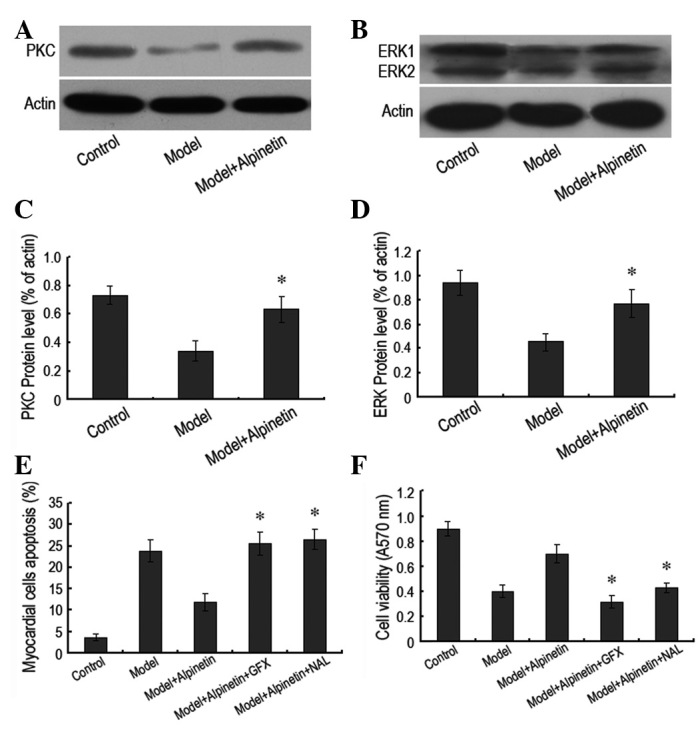
(A–D) Expression levels of intracellular protein kinase C (PKC) and extracellular signal-regulated kinase (ERK) in rat myocardial cells, as analyzed by western blotting following the 48 h culture of rat myocardial cells with serum deprivation and the administration of 120 mg/ml alpinetin. Data shown are the representative results from at least three independent experiments. ^*^P<0.05, versus model group. Analysis of (E) rat myocardial apoptosis using flow cytometry and (F) rat myocardial cell viability using the MTT method following the administration of the PKC antagonist GF109203X (10 μM) or the ERK antagonist U0126 (10 μM), in addition to serum deprivation and alpinetin administration. Data shown are the representative results from at least three independent experiments. ^*^P<0.05, versus model + alpinetin group.

In order to verify the molecular mechanism by which alpinetin protects rat myocardial cells, the signaling pathways of PKC and ERK were studied. Notably, it was observed that following alpinetin administration, the intracellular PKC and ERK protein expression levels increased significantly compared with those in the serum deprivation model (Fig. 3A–D). However, when the PKC inhibitor GF109203X or the ERK inhibitor U0126 was administered to block the corresponding signaling pathways, it was observed that level of myocardial cell apoptosis increased, irrespective of alpinetin administration. Furthermore, the A570 value of the myocardial cells decreased (Fig. 3E and F). These results indicated that the protective effects of alpinetin on the rat myocardial cells were closely associated with the PKC/ERK signaling pathway.

### Downregulating the δ receptor may inhibit the PKC/ERK signaling pathway

**Figure 4:**
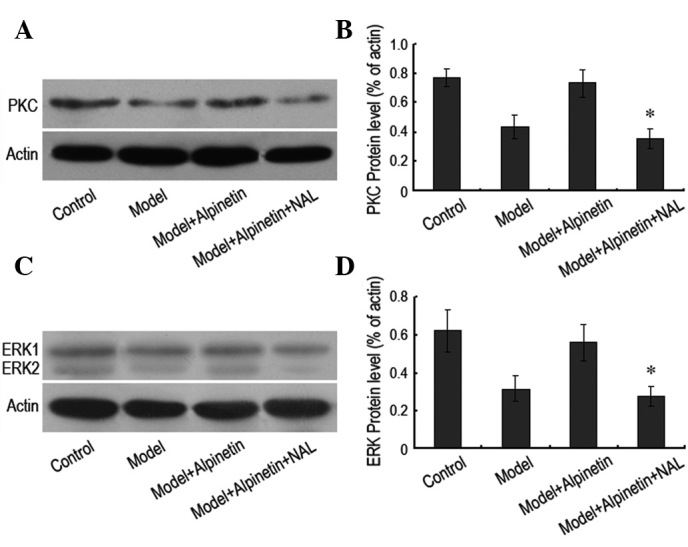
(A–D) Expression levels of intracellular protein kinase C (PKC) and extracellular signal-regulated kinase (ERK) in rat myocardial cells, as analyzed using western blotting following the 48 h culture of rat myocardial cells with serum deprivation and the administration of 120 mg/ml alpinetin, as well as 10 μM naltrindole. Data shown are the representative results from at least three independent experiments. ^*^P<0.05, versus model + alpinetin group.

In order to further demonstrate the interrelation between the δ receptor and the PKC/ERK pathway in the protection of rat myocardial cells, the δ receptor antagonist naltrindole (10 μM) was administered and the expression levels of PKC and ERK protein were assessed. It was observed that the inhibition of the δ receptor resulted in a significant reduction in the intracellular expression levels of PKC and ERK proteins (Fig. 4). This indicated that the δ receptor and the PKC/ERK pathway were important for the protection of rat myocardial cells by alpinetin. Of note was the fact that the function of the δ receptor was upstream of the PKC/ERK pathway.

### Alpinetin inhibits rat myocardial apoptosis via the mitochondrial pathway

**Figure 5:**
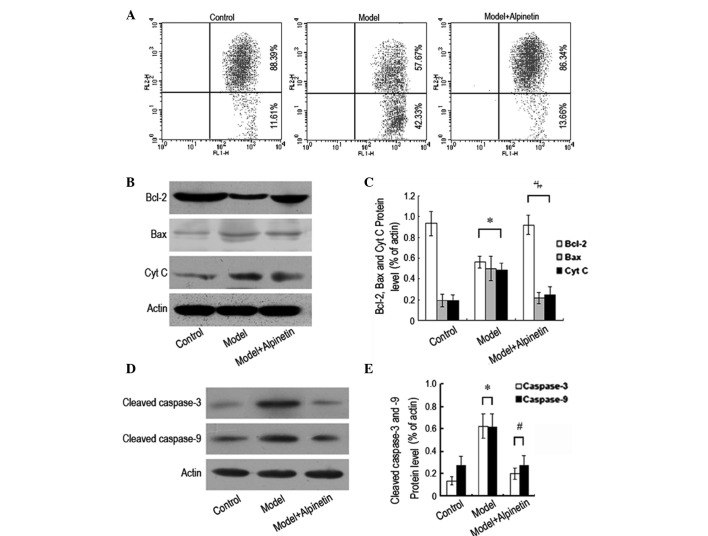
(A) Changes in rat myocardial cell membrane potential analyzed with flow cytometry. Analysis of expression levels of (B and C) Bcl-2, Bax and cytochrome *c (*Cyt *c)* proteins, as well as (D and E) cleaved caspase-3 and cleaved caspase-9 proteins in rat myocardial cells using western blotting following 48 h of serum deprivation with the administration of 120 mg/ml alpinetin. Data shown are the representative results from at least three independent experiments. ^*^P<0.05, versus control group; ^#^P<0.05, versus model group.

In order to further study the molecular mechanism of the rat myocardial apoptosis caused by serum deprivation, flow cytometry and western blotting were used to analyze the changes in the mitochondrial membrane potential and the changes in the expression levels of Bcl-2, Bax and Cyt *c*, respectively. Following 48 h of serum deprivation, the mitochondrial membrane potential of the rat myocardial cells was observed to have decreased, as was the expression level of Bcl-2 protein. However, the expression levels of Bax and Cyt *c* proteins were observed to have increased markedly. Following the administration of alpinetin, the mitochondrial membrane potential of the rat myocardial cells was restored to its original level, the expression level of Bcl-2 protein increased and the expression levels of Bax and Cyt *c* proteins decreased (Fig. 5A–C). In addition, western blotting was used for the analysis of the changes in the expression levels of cleaved caspase-3 and cleaved caspase-9 protein. Following 48 h of serum deprivation, it was observed that the intracellular expression levels of cleaved caspase-3 and cleaved caspase-9 proteins increased significantly. With the administration of alpinetin, the expression levels of the two proteins decreased (Fig. 5D and E). These results indicate that the inhibition of the serum deprivation-induced rat myocardial apoptosis by alpinetin was associated with the mitochondrial pathway.

### Expression of the δ, κ and μ receptors in rat myocardial cells

**Figure 6:**
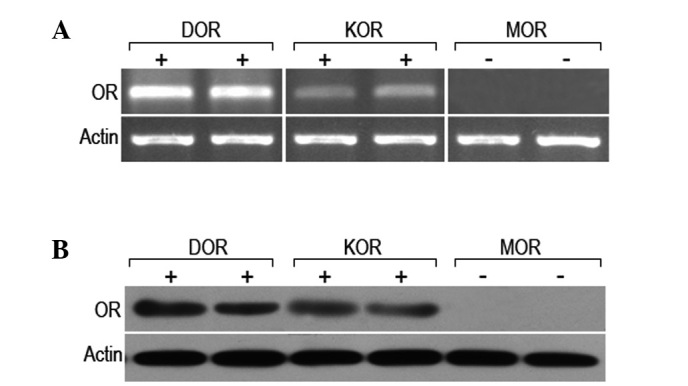
Expression levels of δ, κ and μ opioid receptors (DOR, KOR and MOR, respectively) in rat myocardial cells, as analyzed using (A) reverse transcription-polymerase chain reaction (RT-PCR) and (B) western blotting.

In order to determine the expression levels of the δ, κ and μ receptors in rat myocardial cells, the mRNA and protein levels of the three receptors were analyzed. It was observed that the mRNA and protein of the δ and κ receptors was expressed in the rat myocardial cells; however, no mRNA or protein expression of the μ receptor was detected (Fig. 6). This, in combination with the results from previous studies, indicate that the δ receptor is important in the protective effect of alpinetin in rat myocardial cells.

## Discussion

It has been shown in previous studies that flavonoids are able to protect cells against fatal injury in ischemia-reperfusion, which promotes the proliferation of various cells. This may be used in the treatment of a number of diseases (25–27). As a flavonoid with numerous biological activities, alpinetin has been the subject of much focus globally in recent years, due to its wide distribution and low toxicity.

Apoptosis is important in the regulation of normal organismal development and in the maintenance of the stability of the internal environment. With regard to the cardiovascular system, apoptosis participates in the formation of the heart and blood vessel structure in the early stage of morphogenesis and, at a later stage, regulates the growth and development of the cardiovascular system. Thus, apoptosis is an indispensible process in the development of the cardiovascular system (28). Cell apoptosis and proliferation are coordinated and complement each other to maintain the stability of the internal environment and the normal growth of the body. When the internal environment changes, such a balance may be lost, leading to the onset of disease. The development of studies into cardiovascular diseases has revealed that myocardial apoptosis prevails in the physiological and pathological changes of the cardiovascular system, and is a critical cytological factor in the development of numerous cardiovascular diseases. At present, myocardial apoptosis is considered to be an important factor causing cardiovascular diseases and one of the major reasons leading to a decline in cardiac function (29). Therefore, exploring the mechanism and development of myocardial apoptosis and reducing its occurrence is of great significance in the safeguarding of cardiac structure and function.

It has been demonstrated that apoptosis may be stimulated by a number of techniques, with hydrogen peroxide and ischemia/reaeration used as the common methods to induce myocardial apoptosis. However, the use of strong chemical provocative methods to induce apoptosis may ultimately result in irreversible injury to the cells. In the current study, serum deprivation was used to induce cell apoptosis, which was milder than that induced by the previously mentioned methods and closer to the cell injury caused by ischemia and anoxia *in vivo*. This involved removing the serum from the cell culture medium to maintain the state of serum deprivation, thereby forming the model of the apoptosis and, in addition, alleviating the impact of the varying nature of the serum on the experimental results (30). In this experiment, following 48 h of serum deprivation, the cell proliferation of the cultured myocardial cells was inhibited and apoptosis occurred, which indicated that the growth of the myocardial cells was inhibited.

δ receptors are widely distributed around the body, with numerous receptors existing on the myocardial membrane and blood vessel walls, in addition to the central nervous system (31). In the opioid receptor superfamily, the δ receptor is closely associated with the viability and proliferation of cells (32,33). As demonstrated in previous studies, the activation of the δ receptor promotes the proliferation of rat ventricular muscle cells (34,35). In the current study, alpinetin was administered to rat myocardial cells, leading to an increase in the expression level of the δ receptor, a significant reduction in the myocardial apoptosis rate and an increase in cell proliferation. This indicated that the δ receptor was important in the protection of rat myocardial cells by alpinetin. However, when a specific antagonist to downregulate the δ receptor expression was administered, it was observed that the protective effect of alpinetin in rat myocardial cells disappeared. This indicated that the protective effect of alpinetin was closely correlated with the function and state of the δ receptor.

As demonstrated by previous studies, the δ receptor functions via the G-protein and KATP channel signal transduction pathways (36,37) and is closely associated with PKC (38). PKC is a member of the serine/threonine kinase family and exhibits extensive biological activities, including regulating the proliferation and differentiation of numerous cell types (39–41). It has been suggested that the activation of PKC may induce various cells to proliferate (42), and PKC has been shown to participate in the proliferation and differentiation of myocardial cells. In the present study, it was revealed that alpinetin increased intracellular PKC expression following the serum deprivation of rat myocardial cells. However, when PKC was inhibited, the protective effects of alpinetin on the myocardial cells disappeared. Therefore, it was inferred that the PKC pathway was involved in the protection of the the rat myocardial cells. It has been demonstrated that the participation of PKC in cell proliferation differs according to the various isoforms of the enzyme (43). In a number of experimental models, the different subtypes of PKC have been shown to possess a variety of functions. The complicated nature of the intracellular apoptosis signaling pathways makes it crucial to understand the relationship between the different PKC subtypes.

It was demonstrated in a previous study that the δ receptor acted via the ERK signaling pathway to promote the survival and proliferation of cells (35). In this study, we proposed that alpinetin exerted its protective effects on the rat myocardial cells by exciting the δ receptor to activate PKC, leading to the further activation of ERK. As shown in our results, while the protective effects of alpinetin on the myocardial cells were reversed by a PKC-specific inhibitor, they were also reversed by an ERK-specific inhibitor, U0126. This result supported our previous assumption and was consistent with previous studies (22,23). However, further investigations into the specific mechanism behind the PKC-mediated regulation of ERK are required.

Apoptosis occurs via two pathways, the mitochondrial and death receptor pathways. As demonstrated in a previous study, a δ receptor agonist protected against apoptosis via the mitochondrial pathway (44). In the present study, whether the mitochondrial pathway was relevant to the protective effects of alpinetin on myocardial cells was investigated. It was observed that following alpinetin administration, the mitochondrial membrane potential was restored, the level of Bcl-2 protein in the cytoplasm was increased and the expression levels of Bax and Cyt *c* were decreased. The intracellular expression levels of cleaved caspase-3 and cleaved caspase-9 proteins were also shown to have decreased markedly. It was concluded that the protective effects of alpinetin on rat myocardial cells were elicited via the mitochondrial pathway.

In conclusion, alpinetin protects against rat myocardial cell apoptosis induced by serum deprivation. Furthermore, alpinetin activates the δ receptor to induce the endogenous protection of myocardial cells via the PKC/ERK signaling pathway.
